# Catastrophic health expenditure and associated factors in low-, lower-middle and upper-middle-income countries: systematic review and meta-analysis

**DOI:** 10.1080/16549716.2026.2734678

**Published:** 2026-09-25

**Authors:** Mohammod Akbar Kabir, Maelodee Chong Armstrong, Sayem Ahmed, Jesper Löve, Rumana Huque, Jahangir Khan

**Affiliations:** a School of Public Health and Community Medicine, Institute of Medicine, Sahlgrenska Academy, University of Gothenburg, Gothenburg, Sweden; b Health Economics Research Group (HERG), Department of Health Sciences, Brunel University of London, Uxbridge, UK; c Department of Economics, University of Dhaka, Dhaka, Bangladesh; d Department of Learning, Informatics, Management and Ethics, Karolinska Institutet, Stockholm, Sweden; e Department of International Public Health, Liverpool School of Tropical Medicine, Liverpool, UK

**Keywords:** CHE prevalence, financial risk protection, meta-regression, risk of bias, universal health coverage

## Abstract

**Background:**

Reducing catastrophic health expenditure (CHE) is critical for achieving Universal Health Coverage (UHC), as high out-of-pocket healthcare spending can cause financial hardship and hinder access to care. However, evidence on CHE prevalence and associated factors in low-income (LICs), lower-middle-income (Lower-MICs), and upper-middle-income (UMICs) countries remains fragmented.

**Objectives:**

To systematically review and synthesize evidence on the prevalence and factors of CHE in LICs, Lower-MICs, and UMICs.

**Methods:**

Following registered protocol (PROSPERO: CRD42024521553) and PRISMA guidelines, we searched CINAHL, PubMed, Scopus, and Web of Science for studies published between 2015 and 2023. Quantitative studies that measured CHE and/or associated factors at the 10% threshold of household total expenditure/income and/or the 40% threshold of non-food expenditure were included. Risk of bias was assessed using the AXIS tool. A random-effects meta-analysis estimated the pooled prevalence of CHE, and associated factors were synthesized narratively.

**Results:**

Forty studies were included. Key determinants increasing CHE risk were low income, elderly or chronically ill members, private facilities use, female-headed households, and lack of insurance, while education and employment were protective. Rural residence was generally associated with higher CHE despite mixed findings. Pooled CHE prevalence was 18% (95% CI: 14–23%) at the 10% threshold and 7% (95% CI: 5–10%) at the 40% threshold, with considerable heterogeneity (I^2^ > 99%). UMICs had the highest CHE prevalence, whereas LICs reported the lowest.

**Conclusions:**

CHE remains considerable, particularly in Lower-MICs and UMICs, and varies across income groups. Achieving UHC requires policies to strengthen financial protection and reduce socioeconomic disparities.

## Paper Context

- **Main findings**: Catastrophic health expenditure is highly prevalent and varies across settings, driven by socioeconomic status, female-headed households, elderly/chronically ill members, and private facility use. Education and employment are protective. Rural residence was more consistently associated with a higher risk of CHE, despite mixed findings.
- **Added knowledge**: This review provides consolidated evidence that the burden of catastrophic health expenditure is sustained with varying degrees in lower-, lower-middle-, and upper-middle-income countries, with the highest burden in upper-middle-income countries, and that economic growth alone does not prevent financial risk from catastrophic health expenditure, while highlighting key determinants.
- **Global health impact for policy and action**: Context-specific Policy efforts targeting subsidies for vulnerable groups, regulating healthcare costs, and strengthening public health systems can significantly reduce the risk of CHE and advance universal health coverage.

## Background

Protecting households from financial risk when accessing healthcare services is a key policy goal of healthcare systems worldwide [1]. Financial risk protection (FRP) is achieved when: (i) services for healthcare are attainable without any financial barriers; and (ii) direct costs involved in accessing healthcare are not a cause of financial disaster [2]. The World Health Organization (WHO), in its World Health Report, highlighted the importance of universal health coverage (UHC), meaning that all individuals should have access to healthcare services as needed, without incurring financial hardship [1]. Thus, FRP is one of the key components of UHC, and ensuring it should be key function of all healthcare systems. Although some countries achieved substantial progress in FRP, it remains a challenge for many, especially in low- and middle-income countries (LMICs). Along with UHC, FRP has gained great attention among policymakers and researchers following the publication of the WHO’s World Health Report in 2010. This attention has further increased since the United Nations adopted Sustainable Development Goals (SDGs) in 2015, with the UHC target 3.8, including coverage of healthcare services (SDG 3.8.1) and FRP (SDG 3.8.2) [3].

Catastrophic health expenditure (CHE), defined as Out-Of-Pocket (OOP) healthcare payments exceeding a specified threshold, is a key indicator of FRP and inequity in healthcare access [4,5]. In many low-income (LICs), lower-middle-income (Lower-MICs), and upper-middle-income (UMICs) countries, where public health financing remains inadequate and private spending on healthcare is high, CHE can push households into poverty, force them to forgo necessary care, or lead to the sale of assets, perpetuating cycles of poverty and inequality [6].

Based on OOP spending, two definitions are commonly used to estimate CHE in healthcare. Firstly, the portion of total household spending [7], and secondly, the portion of the household’s total non-food spending [8,9]. Studies have used different thresholds, including 10%, 25%, and 40%, to estimate the CHE prevalence [7,8]. Such methodological heterogeneity complicates meaningful comparisons of CHE estimations across settings [4]. The 10% threshold is used by the World Health Organization and the World Bank to monitor FRP under UHC, while the 40% threshold remains widely used in the literature within the capacity-to-pay framework. Therefore, this review focused on the 10% and 40% thresholds, as they represent the two most common and conceptually distinct approaches for measuring CHE.

As reducing the prevalence of CHE and ensuring FRP in healthcare are key policy goals across the healthcare systems globally, and FRP is vital to achieving UHC in LICs, Lower-MICs, and UMICs, understanding both the prevalence of CHE and its determinants is important for designing effective healthcare policies and FRP strategies. Despite its policy importance, the current evidence on CHE prevalence and its associated factors remains fragmented, often limited to individual countries or regions. To date, no comprehensive systematic review and meta-analysis have synthesized CHE prevalence and associated factors across LICs, Lower-MICs, and UMICs using both 10% and 40% thresholds. This limits a clearer, more complete understanding of CHE prevalence and associated factors, which is critical for deriving generalizable, actionable policy recommendations to achieve UHC with FRP. To address this evidence gap, this review was guided by the following research questions:

1. What is the prevalence of CHE among households in low-, lower-middle- and upper-middle-income countries? and
2. What factors are associated with CHE in low-, lower-middle-, and upper-middle-income countries?

Accordingly, this systematic review and meta-analysis aimed to synthesize the available literature on CHE prevalence and its associated factors across LICs, Lower-MICs, and UMICs.

## Methods

This review followed the protocol registered with PROSPERO (CRD42024521553) available at https://www.crd.york.ac.uk/PROSPERO/view/CRD42024521553. The findings were reported following the guidelines of the Preferred Reporting Items for Systematic Reviews and Meta-Analyses (PRISMA) [10].

### Search strategy

Electronic databases, namely CINAHL, PubMed, Scopus, and Web of Science, were searched to identify relevant studies. FRP, along with UHC, is a key health-related target of the United Nations’ SDGs adopted in 2015. In view of the healthcare goal of this SDGs, the literature was searched on 30 November 2023, covering the period from 30 September 2015 to 30 November 2023. This review included English language studies only. Reference lists of included studies and published reviews on relevant topics were also manually searched to ensure the comprehensiveness of the list. Eligible articles identified from the reference lists were accessed as an additional data source. The standard Google search engine was used to find the full text of articles identified from the reference lists. Keywords including ‘influencing factors’, ‘factors associated with’, ‘Determinants’, ‘Catastrophic Health Expenditures’, ‘costs’, ‘financial catastrophe’, ‘Financial Protection’, ‘risk factors’, ‘health care’, and the name of LICs, Lower-MICs and UMICs countries classified in 2023 based on countries per capita income by World bank were used as search terms. The Boolean operators ‘AND’ and ‘OR’ were also used to combine the search terms and extend the search. The detailed search string is shown in Table S1 of the supplementary file.

### Selection of studies

The selection of studies was performed in several steps. At the beginning, the review team’s two members conducted the database search in November 2023 using an appropriate strategy, and the results were imported into EndNote. After duplicates were removed, two independent reviewers screened the remaining studies in two stages. In the first stage, the titles and abstracts were reviewed, and the available full texts of the selected studies were then downloaded and assessed for inclusion. All observational quantitative studies that measured the CHE prevalence or identified its associated factors using country-level household data in LIC, Lower-MIC, and UMIC settings, following the definition of 10% threshold of household total income or expenditure, and/or 40% threshold of household non-food expenditure, were considered eligible for inclusion. To reflect the increased global attention to FRP following the WHO World Health Report 2010, only studies using household survey data collected from 2010 onward were eligible. Detailed eligibility criteria for inclusion studies are shown in Table S2 of the supplementary file. Disputes in the assessment were resolved through discussion among the reviewers. There was a plan to involve a third reviewer if they could not reach 100% agreement.

### Data extraction method and data items

After the screening process, two reviewers independently extracted data from the selected full-text articles for the following items: study author(s), study title, year of publication, study location (country), country’s income group, population, sample size (household), study design, rate of CHE at the 10% threshold level of household total income/expenditure, lower and upper limits of the confidence interval (CI) of CHE estimate at the 10% threshold, standard error (SE) of CHE estimate at the 10% threshold, rate of CHE at the 40% threshold level of household non-food expenditure, lower and upper limits of the CI of CHE estimate at the 40% threshold, SE of CHE estimate at the 40% threshold, funding source, competing interests, and factors associated with CHE in healthcare as reported from the analysis of statistical models. Unreported SE were calculated from available data. For studies reporting prevalence and sample size, SE were derived using the formula $\mathrm{SE}=\mathrm{sqrt}\left[p\left(1-p\right)/n\right],$ where p is the prevalence proportion and n is the sample size. For studies reporting CI, SEs were calculated as (upper CI – lower CI)/(2 × 1.96). The extracted data were organized into an Excel sheet, and disagreements were resolved through discussion until 100% agreement was reached.

### Risk of bias assessment

As the included studies were cross-sectional, observational, and quantitative, the reporting quality and risk of bias were assessed by two independent reviewers using the AXIS assessment tool [11]. This approach is consistent with other recent systematic reviews assessing cross-sectional evidence [12]. The appraisal tool consists of 20 items of which seven (items 1, 4, 10, 11, 12, 16 and 18) are linked with reporting quality, seven (items 2, 3, 5, 8, 17, 19 and 20) are linked to study design and six (items 6, 7, 9, 13, 14 and 15) are linked to the possibility of biases. The assessment tool is detailed in the supplementary file (Table S3). The authors classified the studies’ quality as high if they met 70% of the criteria, medium if they met 40% to 69%, and low if they met < 40%. Similar score-based categorizations have been applied in previous systematic reviews using the AXIS tool [13]. Publication bias was judged by checking funnel plot asymmetry, and the result was confirmed using Egger’s test of the regression line. Publication bias was further assessed using Doi plots with the Luis Furuya-Kanamori (LFK) index. LFK index values between −1 and +1 indicate no asymmetry, between ±1 and ±2 indicate minor asymmetry, and exceeding ±2 indicate major asymmetry.

### Certainty of evidence assessment

The certainty of evidence for the pooled prevalence estimates was assessed using the Grading of Recommendations Assessment, Development and Evaluation (GRADE) approach adapted for prevalence studies [14]. Evidence was assessed across five domains that may lead to downgrading: risk of bias, inconsistency, indirectness, imprecision, and publication bias. Inconsistency was assessed using between-study heterogeneity, indirectness based on the relevance of study populations and outcome definitions, and imprecision by sample size and CIs. The overall certainty was rated as high, moderate, low, or very low.

### Strategy for data synthesis

Data were summarized using descriptive statistics and described in narrative form. The number of cases was calculated from the sample size and the CHE prevalence rate for analysis. Heterogeneity was assessed using Cochran’s Q and the I^2^ statistic and was considered significant when the Q test P−value<0.10 or I>50% [15]. The DerSimonian-Laird random-effects model was used for the meta-analysis when the heterogeneity test was significant. The model accommodates between-study variability and incorporates study sample size through inverse-variance weighting, whereby studies with greater precision receive relatively larger weights in the pooled estimates. Subgroup analysis was performed across LIC, Lower-MIC, and UMIC to explore potential causes of heterogeneity and was investigated using a forest plot. We adopted this World Bank income classification because national income level is closely linked to health financing arrangements, public health expenditure, insurance coverage, and broader social protection mechanisms. Similar income-based comparisons have been adopted in multi-country analyses of CHE and UHC monitoring reports [4,16]. We also performed random-effects meta-regression to examine whether study-level characteristics, including publication year, income group, and study quality, explained between-study heterogeneity. Several countries contributed more than one study, which may have disproportionately influenced the pooled estimates, particularly when studies from the same country reported relatively high or low prevalence estimates. To assess the potential impact of this unequal representation, a sensitivity analysis was performed using the latest study for each country, thereby reducing within-country clustering and evaluating the robustness of our pooled estimates to country-level oversampling. Stata software (version 14.1) was used for data analysis. Factors associated with CHE were extracted and synthesized using a conceptual framework comprising six themes, including household head characteristics, household characteristics, household member characteristics, social protection characteristics, health system characteristics, and sociocultural factors (Figure 1). The framework was developed based on themes from previous systematic reviews on CHE determinants [17,18] and refined to reflect the factors identified in the included studies, which emphasize the interplay among socioeconomic, demographic, health need, social protection, and health system factors in shaping financial risk protection outcomes.

**Figure 1.**
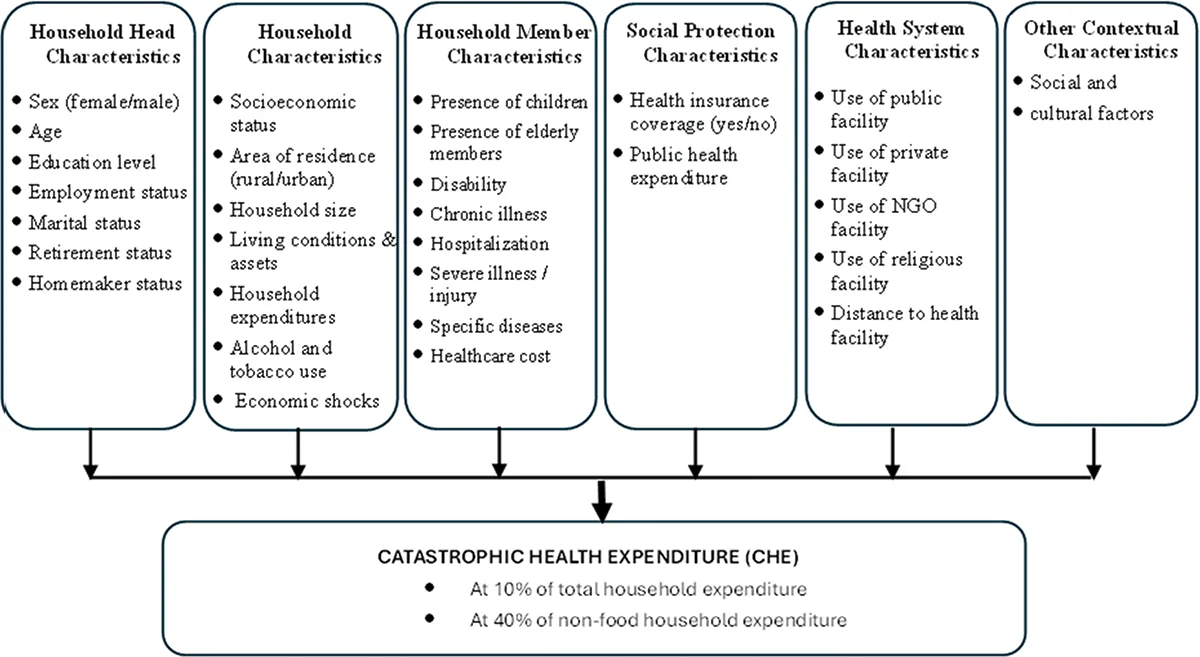
Conceptual framework for factors of catastrophic health expenditure.

## Results

### Study characteristics

The review identified 1479 articles from the databases (287 articles from CINAHL, 359 from PubMed, 446 from Scopus, and 387 from Web of Science). After removing 689 duplicates, 790 titles and abstracts were screened, and 62 full-text articles were retrieved and assessed for eligibility; 35 [19–53] met the inclusion criteria and were included in the review (Figure 2). Twenty‑seven studies [54–80] were excluded because they failed to meet the eligibility criteria. Of the 27 studies, 5 were excluded due to not reporting the CHE measure (PICO) [64,67,69,72,73], 11 were excluded due to study design including use of wrong threshold and not using national level data [56,61,63,65,68,71,74–78], 11 were excluded due to study type and other reasons including non-English publication, use of data collected before 2010, insufficient information and reporting correction only [54,55,57–60,62,66,70,79,80]. This review also included 5 more studies [81–85] after assessing the eligibility criteria of 11 articles identified in the reference lists of the selected studies [86–91]. Six studies were excluded due to insufficient information and outdated data [9,92–96]. A total of 40 studies were selected for this review to synthesize data. Table 1 presents the characteristics of included studies from LICs, Lower-MICs, and UMICs (World Bank classification [97]), with sample sizes ranging from 2,282 to 113,823 households.

**Figure 2.**
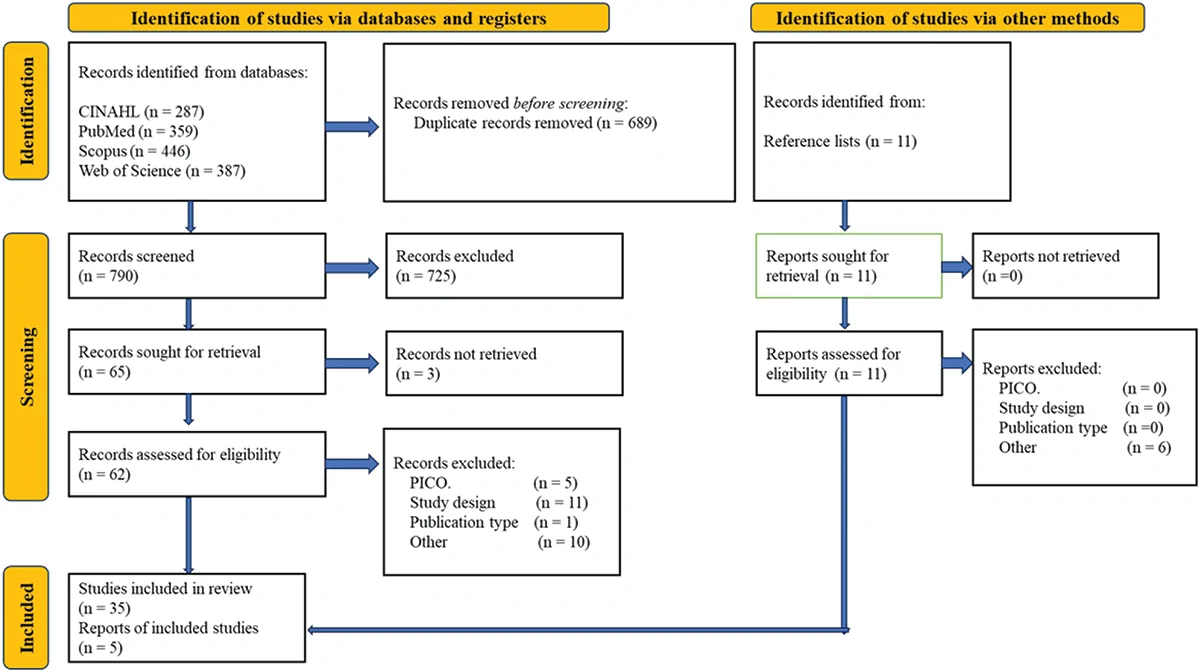
PRISMA flow diagram for study selection process. PRISMA: preferred reporting items for systematic reviews and meta-analyses.

**Table 1.** Study characteristics.

SL	Study Author(s)	Year of Publication	Study Country	Country’s Income group	Sample Size	Incidence of CHE^a^	Incidence of CHE^b^	Funded	Competing interests	Study Quality
1	Ahmed, S., Ahmed, M. W., Hasan, M. Z., Mehdi, G. G., Islam, Z., Rehnberg, C., Niessen, L. W., and Khan, J. A. M.	2022	Bangladesh	Lower-middle income	46,076	24.60	10.90	No	No competing interests	High
2	Sharma, J., Pavlova, M., and Groot, W.	2023	Bhutan	Lower-middle income	11,660	–	1.51	Not declared	Not declared	High
3	Attia-Konan, A. R., Oga, A. S. S., Kouame, J., Alla, A. E. H., Koffi, K., and Kouadio, L.	2020	Côte d’Ivoire	Lower-middle income	12,899	–	4.02	Not declared	No competing interests	Moderate
4	Cros, M., Cavagnero, E., Alfred, J. P., Sjoblom, M., Collin, N., and Mathurin, T.	2019	Haiti	Lower-middle income	2,282	11.54	–	No	No competing interests	High
5	Mohanty, S. A. N. J. A. Y. K., Kim, R. O. C. K. L. I., Khan, P. I. J. U. S. H. K. A. N. T. I. and Subramanian, S. V.	2018	India	Lower-middle income	101,576	–	23.40	Not declared	Not declared	Moderate
6	Dwivedi, R., Pradhan, J., and Athe, R.	2021	India	Lower-middle income	101,662	17.32	4.04	Not declared	No competing interests	Moderate
7	Gaddam, R. and Rao, K. R.	2023	India	Lower-middle income	113,823	16.69	–	Yes	No competing interests	High
8	Yazdi-Feyzabadi, V., Bahrampour, M., Rashidian, A., Haghdoost, A. A., Akbari Javar, M. and Mehrolhassani, M. H.	2018	Iran	Lower-middle income	38,148	–	3.25	No	No competing interests	High
9	Joshani Kheibari, M., Esmaeili, R. and Kazemian, M.	2019	Iran	Lower-middle income	38,146	–	3.45	Yes	No competing interests	Moderate
10	Yazdi-Feyzabadi, V., Mehrolhassani, M. H., and Darvishi, A.	2019	Iran	Lower-middle income	37,866	–	3.46	Yes	No competing interests	High
11	Rezaei, S. and Hajizadeh, M.	2019	Iran	Lower-middle income	37,860	–	5.26	Yes	No competing interests	High
12	Kazemi-Karyani, A., Woldemichael, A., Soofi, M., Karami Matin, B., Soltani, S. and Yahyavi Dizaj, J.	2020	Iran	Lower-middle income	37,959	–	3.32	Yes	No competing interests	High
13	Kimani, D.N., Mugo, M.G. and Kioko, U.M.	2016	Kenya	Lower-middle income	8,844	14.35	9.84	Not declared	Not declared	Moderate
14	Barasa, E. W., Maina, T., and Ravishankar, N.	2017	Kenya	Lower-middle income	33,675	–	6.58	Yes	No competing interests	High
15	Nundoochan, A., Thorabally, Y., Monohur, S., and Hsu, J.	2019	Mauritius	Lower-middle income	6,720	8.85	1.25	Yes	No competing interests	Moderate
16	Oudmane, M., Mourji, F., and Ezzrari, A.	2019	Morocco	Lower-middle income	15,970	12.80	1.77	No	Not declared	Moderate
17	Ghimire, M., Ayer, R., and Kondo, M.	2018	Nepal	Lower-middle income	5,988	–	10.30	No	No competing interests	Moderate
18	Aregbeshola, B.S., and Khan, S.M.	2018	Nigeria	Lower-middle income	38,700	16.40	13.70	Not declared	No competing interests	Moderate
19	Cleopatra, I., and Eunice, K.,	2018	Nigeria	Lower-middle income	4,581	–	35.99	Not declared	Not declared	Moderate
20	Edeh, H. C.	2022	Nigeria	Lower-middle income	5,000	47.64	–	Yes	No competing interests	High
21	Bashir, S., Kishwar, S., and Salman	2021	Pakistan	Lower-middle income	24,019	13.15	4.57	No	No competing interests	Moderate
22	Farooq, S. and Masud, F.	2021	Pakistan	Lower-middle income	4,023	22.40	15.90	Not declared	Not declared	Moderate
23	Séne, L. M., and Cissé, M.	2015	Senegal	Lower-middle income	5,953	6.26	–	Not declared	Not declared	Moderate
24	Ayadi, I., and Zouari, S.	2017	Tunisia	Lower-middle income	11,291	17.12	–	Yes	No competing interests	Moderate
25	Ismaïl, S., and Arfa, C.	2022	Tunisia	Lower-middle income	25,087	18.36	2.22	No	No competing interests	High
26	Dastan, I., Abbasi, A., Arfa, C., Hashimi, M. N. and Alawi, S. M. K.	2021	Afghanistan	Low-income	19,838	31.69	13.53	No	No competing interests	High
27	Kiros, M., Dessie, E., Jbaily, A., Tolla, M.T., Johansson, K.A., Norheim, O.F., Memirie, S.T. and Verguet, S	2020	Ethiopia	Low-income	30,229	2.10	0.41	Yes	No competing interests	High
28	Zewde, I. F., Kedir, A. and Norheim, O. F.	2023	Ethiopia	Low-income	5,900	4.70	3.10	No	Not declared	Moderate
29	McHenga, M., Chirwa, G. C. and Chiwaula, L. S.	2017	Malawi	Low-income	12,271	–	0.73	No	No competing interests	High
30	Mulaga, A. N., Kamndaya, M. S. and Masangwi, S. J.	2021	Malawi	Low-income	12,447	4.14	1.34	Yes	No competing interests	High
31	Mulaga, A. N., Kamndaya, M. S. and Masangwi, S. J.	2022	Malawi	Low-income	12,447	–	1.34	Yes	No competing interests	High
32	Edoka, I., McPake, B., Ensor, T., Amara, R. and Edem-Hotah, J.	2017	Sierra Leone	Low-income	6,800	32.00	–	Yes	No competing interests	High
33	Kwesiga, B., Aliti, T., Nabukhonzo, P., Najuko, S., Byawaka, P., Hsu, J., Ataguba, J. E. and Kabaniha, G.	2020	Uganda	Low-income	17,320	14.20	–	Yes	No competing interests	High
34	Ma, X., Wang, Z., and Liu, X.,	2019	China	Upper-middle income	13,884	25.09	8.94	Not declared	Not declared	High
35	Liu, C., Liu, Z. M., Nicholas, S. and Wang, J.	2021	China	Upper-middle income	11,820	–	8.70	Yes	No competing interests	High
36	Jia, Y. S., Hu, M., Fu, H. Q. and Yip, W.	2022	China	Upper-middle income	40,011	28.90	11.00	Yes	No competing interests	High
37	Li, Y., Guan, H., and Fu, H.	2023	China	Upper-middle income	9,728	36.18	17.73	Yes	No competing interests	High
38	Taniguchi, H., Rahman, M. M., Swe, K. T., Islam, M. R., Rahman, M. S., Parsell, N., Hussain, A., Shibuya, K., and Hashizume, M.	2021	Iraq	Upper-middle income	24,944	3.30	2.20	Yes	No competing interests	High
39	Falconi, D. P. and Bernabé, E.	2018	Peru	Upper-middle income	30,966	–	4.09	Not declared	Not declared	Moderate
40	Özgen Narcı, H., Şahin, İ. and Yıldırım, H. H.	2015	Turkey	Upper-middle income	42,886	–	0.75	Yes	No competing interests	High

^a^Incidence of CHE at 10% level of the household’s total expenditure.

^b^Incidence of CHE at 40% level of non-food expenditure.

Of the included studies, eight [19,26,38–40,49,53,82] from LICs, twenty-five [20,21,25,27–35,37,41–46,48,50,51,83–85] from Lower-MICs, and seven from UMICs [22–24,36,47,52,81] on CHE issues. Twenty‑four studies reported CHE incidence at 10% threshold [19,20,22,23,26,27,29,30,36,39,41,42,44–46,48–51,53,81–84], ranging from 2.10% in Ethiopia [82] to 47.64% in Nigeria [44], and 33 reported CHE incidence at 40% threshold [19–26,28,29,31–43,45–47,50,52,81–85], ranging from 0.41% in Ethiopia [82] to 35.99% in Nigeria [85].

Of the 40 reviewed studies, 19 declared that they received funds [22–24,30–34,36,37,39–41,44,49,51–53,82] and 12 studies not disclosing funding information [21,25,28,29,35,46–48,81,83–85]; 30 declared no competing interest [19,20,22–25,27,29–41,43–45,49–53,82,84] and 10 did not mention anything [21,26,28,42,46–48,81,83,85]; 16 were assessed as moderate quality [25,26,28,29,32,41–43,45–48,51,83–85] and 24 as high quality [19–24,27,30,31,33–40,44,49,50,52,53,81,82].

### Determinants of financial risk from CHE

We identified associated factors of CHE for 10% of households’ total expenditure and 40% of non-food expenditure and synthesized the results using a conceptual framework of 6 broad themes, presented in Tables 2 and 3, respectively, with narrative synthesis. The detailed counts of studies that identified each significant factor are presented in Tables S4 and S5 of the supplementary file.

**Table 2.** Narrative synthesis of factors associated with CHE at the 10% threshold of total household expenditure.

Domain	Key Determinants	Overall Association with CHE	Pattern Across Income Groups
Household Head Characteristics	Sex, age, education, literacy, employment, marital status, and retirement status, homemaker status	Evidence was mixed for sex and marital status. Older age is generally associated with higher CHE prevalence, whereas higher education, literacy, and employment are associated with lower CHE prevalence. Retirement and homemaker status were associated with increased CHE in a few studies.	Protective effects of education, literacy, and employment were predominantly reported in Lower-MICs and UMICs, while associations for sex and age varied across settings. Older households with a higher prevalence of CHE and female-headed households with a lower prevalence of CHE were more frequently reported in LICs and Lower-MICs, respectively. Evidence from Lower-MICs was comparatively higher.
Household Characteristics	Area of residence, socioeconomic status, poverty, household size, total expenditure, health expenditure (inpatient and outpatient service), living conditions, alcohol and tobacco use, and economic shocks	Rural residence, poverty, economic shocks, and higher expenditure patterns generally increased CHE, whereas higher socioeconomic status and better living conditions were consistently associated with reduced CHE prevalence. larger household size.	Rural disadvantage was consistently associated with CHE across all income groups, particularly in Lower-MICs and UMICs. The protective effects of higher socio-economic status and better living conditions were stronger in Lower-MICs and UMICs. Economic shocks were mainly reported in LICs, whereas inpatient and outpatient expenditures were more prominent in Lower-MICs and UMICs. The effects of larger household size were more consistent in LICs with increased CHE and in Lowe-MICs with reduced CHE.
Household Member Characteristics	Children, elderly members, disability, chronic illness, hospitalization, and specific diseases	The presence of elderly members, chronic illness, disability, hospitalization, and specific diseases is consistently associated with increased CHE. Evidence regarding children and disability was mixed.	The effect of elderly household members and chronic illness was the most consistent factor across all income groups, especially in Lower-MICs. Disability with increased CHE was more consistent across Lower-MICs and UMICs. Hospitalization and the presence of children with increased CHE were reported in LICs and Lower-MICs, while disease-specific conditions were mainly reported in LICs.
Social Protection Characteristics	Health insurance coverage, public health expenditure	Health insurance coverage and greater public health expenditure are generally associated with a reduced likelihood of CHE.	The effects of insurance were predominantly observed in Lower-MICs, while evidence on public health expenditure was limited to UMICs.
Health System Characteristics	Type of healthcare facility utilized, choice of private facility uses, distance to health facilities, and primary care use	Utilization of private healthcare facilities and greater distance to health facilities were associated with increased CHE, whereas access to public, NGO, religious, and primary healthcare services was generally associated with reduced CHE.	Higher CHE associated with private healthcare utilization and distance to healthcare facility was consistently reported in Lowe-MICs, while evidence on primary care and NGO facilities with reduced CHE was confined to Lower-MICs settings. The association with the use of religious healthcare facilities was reported in LICs, whereas public healthcare facilities were associated with increased CHE prevalence in both LICs and Lower-MICs.
Sociocultural Factors	Religion, social group	Social and contextual factors showed varied associations with CHE.	These factors were largely context-specific and predominantly reported in Lower-MICs.

**Table 3.** Narrative synthesis of factors associated with CHE at the 40% threshold of non-food expenditure.

Domain	Key Determinants	Overall Association with CHE	Pattern Across Income Groups
Household Head Characteristics	Sex, age, education, literacy, employment, marital status, and retirement status, homemaker status	Female-headed households, older age, retirement, marital status, and homemaker status were associated with higher CHE, whereas literacy, higher educational attainment, and employment were consistently associated with reduced CHE.	The protective effects of education, literacy, and employment were most evident in Lower-MICs and UMICs. Increased CHE among female-headed and older age-headed households was mainly reported in Lower-MICs, while evidence from LICs remained comparatively limited.
Household Characteristics	area of residence, socioeconomic status, household size, health expenditure (inpatient and outpatient service), living conditions, and economic shocks	Rural residence, economic shocks, and higher health expenditure generally increased CHE, whereas higher socioeconomic status and better living conditions were consistently associated with reduced CHE prevalence. Evidence on the association between household size and CHE was mixed.	Rural disadvantage was consistently associated with CHE across all income groups, particularly in Lower-MLICs. The protective effects of higher socioeconomic status and better living conditions were more reported in Lower-MICs and UMICs. Economic shocks were mainly reported in LICs, whereas inpatient and outpatient expenditures were more prominent in Lower-MICs and UMICs. The effects of larger household size were more consistent in Lowe-MICs and UMICs with reduced CHE.
Household Member Characteristics	Children, elderly members, disability, chronic illness, severe illness/injury, hospitalization, and specific diseases	The presence of elderly members, disability, chronic illness, severe illness, and hospitalization was consistently associated with increased CHE. Evidence regarding the presence of children was mixed.	The presence of elderly members and chronic illness were the most consistent factors across Lower-MICs and UMICs, with these findings reported more frequently in Lower-MICs. Hospitalization was consistent across LICs and Lower-MICs. The effect of children and severe illness was reported mainly in LICs and Lower-MICs, whereas disease-specific conditions were reported mainly in LICs.
Social Protection Characteristics	Health insurance coverage, public health expenditure	Health insurance coverage and greater public health expenditure are generally associated with a reduced likelihood of CHE.	Protective effects of health insurance and public health expenditure were consistent in UMICs, whereas the effect of health insurance in Lower-MICs was mixed, with protection reported in most countries where it was reported.
Health System Characteristics	Type of healthcare facility utilized, choice of private facility uses, distance to health facilities, and utilization	Utilization of private healthcare facilities and greater distance to health facilities were associated with increased CHE, whereas access to public, NGO, and primary healthcare services was generally associated with reduced CHE.	The association of Higher CHE with private, public, and healthcare utilization, and with distance to health facilities, was consistently reported in Lower-MICs, whereas evidence of an association with religious facilities and increased CHE was reported in LICs. Access to NGO facilities was associated with reduced CHE, mainly observed in Lower-MIC settings.
Sociocultural Factors	Religion	Religion exhibited a heterogeneous association with CHE.	This association was reported only in Lower-MICs.

### Household head characteristics

Across both CHE thresholds, higher educational attainment or literacy [19–22,28,29,41–43,45,46,48,50,52] and employment status [19,21,29,37,44,45,47,51,85] were consistently associated with a lower likelihood of CHE, particularly in Lower-MICs and UMICs. In contrast, older age of the household head was generally associated with increased CHE prevalence, with stronger evidence from LICs [19,39] at the 10% threshold, and predominantly from Lower-MICs at the 40% threshold [20,31,37,45,85]. Associations with gender and marital status were heterogeneous across studies; female-headed households were predominantly associated with lower CHE prevalence in Lower-LMICs at the 10% threshold [45,50,51], but were more consistently associated with higher CHE prevalence at the 40% threshold across all income groups [19,22,28,31,42,46]. Retirement and homemaker status of household heads were identified as additional risk factors of CHE prevalence in a small number of studies, primarily in Lower-MICs [41].

### Household characteristics

Household-level socioeconomic conditions emerged as important factors in CHE at both the 10% and 40% thresholds. Rural residence [19,21–23,28–31,35–37,39,42,46–52], and higher healthcare expenditures (inpatient and outpatient) [22,45] were consistently associated with increased CHE prevalence, whereas higher socioeconomic status [19–22,27,29,30,35,37,42–44,46,48,51,52,81,85] and better living conditions were protective [25,29,46]. Rural disadvantage was evident across all income groups but most frequently reported in Lower-MICs [21,28,29,31,35,37,42,46,48,50,51], although a few reported urban disadvantages associated with higher CHE prevalence [41,45]. Economic shocks associated with a higher prevalence of CHE, reported mainly in LICs [19]. The association between household size and CHE varied across thresholds and settings; larger household size tended to increase CHE in LICs [39,49,53] at 10% threshold, but showed a more protective association in Lower-MICs [20,25,29,31,45,50] and UMICs at both thresholds. Higher total expenditure also increased the prevalence in Lower-MIC households at 10% threshold [51].

### Household member characteristics

The presence of elderly household members, chronic illness, disability, hospitalization, and severe illness was repeatedly associated with increased CHE at both 10% and 40% thresholds. Elderly members were most strongly associated with CHE prevalence, particularly in Lower-MICs [20,21,27,29,30,35,37,41–43,45,46,50,51], although similar patterns were observed in UMICs [22,36,47,52]. Chronic illness was consistently associated with increased CHE in LICs [39,49] at the 10% threshold, in UMICs [47] at 40% threshold, and in Lower-MICs [20,25,37,43,46,50] at both thresholds. Hospitalization was consistently associated with higher CHE in LICs [39] and Lower-MICs [42,46,85] at both thresholds, whereas disease-specific conditions, including infectious, respiratory, and circulatory diseases, were primarily reported in LICs [19]. Evidence on the association between the presence of children and CHE prevalence was mixed, with cases mainly reported in LICs [52] and Lower-MICs [20,21,29,42,50,51,53]. Disability with higher CHE prevalence was consistent across Lower-MICs and UMICs [37,50,52].

### Social protection characteristics

Health insurance coverage and higher levels of public health expenditure were associated with CHE prevalence at both thresholds, although the consistency of these effects varied by income group. The association between insurance status and CHE was predominantly observed in Lower-MICs, with mixed effects at both thresholds [27,30,31,42,48], whereas evidence was reported only in UMICs with consistently protective effects [47,52]. Evidence on public health expenditure was limited but protective and consistently reported in UMICs at both thresholds [22].

### Health system characteristics

Several studies evaluated the association between health system characteristics and the prevalence of CHE. Utilization of private [20,27], public [20,27,49], and religious health facilities, and greater distance to health facilities [46], were associated with increased CHE, whereas access to NGO [20] and primary healthcare services [48] was generally associated with lower CHE. The adverse effects of private healthcare utilization were most consistently reported in Lower-MICs [20,27], whereas utilization of NGO facilities [20] and primary care services [48] emerged as mitigating factors confined to Lower-MIC settings. Higher CHE was consistently associated with the use of public facilities in both LICs [49] and Lower-MICs [20,27] at the 10% threshold, and in MICs at the 40% threshold [20].

### Other sociocultural factors

Sociocultural factors, including religion and social group affiliation, were examined in a very few studies and reported entirely from Lower-MICs [29,30]. Compared with Hindu households, Muslim and Christian households showed higher CHE prevalence at the 10% threshold [30], while households belonging to other religious groups (e.g. Sikhism and Jainism) showed mixed associations, with higher CHE at the 10% threshold and lower CHE at the 40% threshold. Similarly, social group affiliation showed heterogeneous effects: households from tribal and caste groups at the 10% threshold [30] and other social categories at both thresholds generally reported lower CHE prevalence than the general population.

### Prevalence of CHE at 10% threshold of the household’s total expenditure

The forest plot in Figure 3 presents the pooled CHE estimate overall and by income groups at 10% threshold of households’ total expenditure. The results showed considerable between-studies heterogeneity $\left(I^{2}>99\%,\tau^{2}=0.01,p<0.001\right)$. The estimate of 18% (95% CI: 13.0–22.0) indicates significant financial risk from CHE across populations, though with substantial variability among studies, ranging from 2% in Ethiopia [82] to 48% in Nigeria [44]. Egger’s regression test revealed strong evidence of publication bias (bias coefficient = 45.63, *p* = 0.006), suggesting potential small-study effects [98]. Asymmetry in the Funnel plot (Figure 4) further suggested publication bias. Consistent with these findings, the DOI plot with LFK index 2.76 exhibited major asymmetry (Figure S1 in the supplementary file), indicating substantial small-study effects. Together, these results highlight the need for caution when interpreting the pooled estimates. We conducted sub-group analysis across LIC, Lower-MIC and UMIC to explain heterogeneity and found the pooled prevalence in LICs (6 studies of 5 countries) [19,26,39,49,53,82] as 15.0% (95% CI: 4.0–26.0), in Lower-MICs (14 studies of 12 countries) [20,27,29,30,41,42,44–46,48,50,51,83,84] as 18.0% (95% CI: 13.0–23.0) and in UMICs (4 studies of 2 countries) [22,23,36,81] as 23.0% (95% CI: 10.0–37.0). Variability in CHE prevalence is evident across groups, with Lower-MICs showing tighter confidence intervals, possibly due to more consistent data.

**Figure 3.**
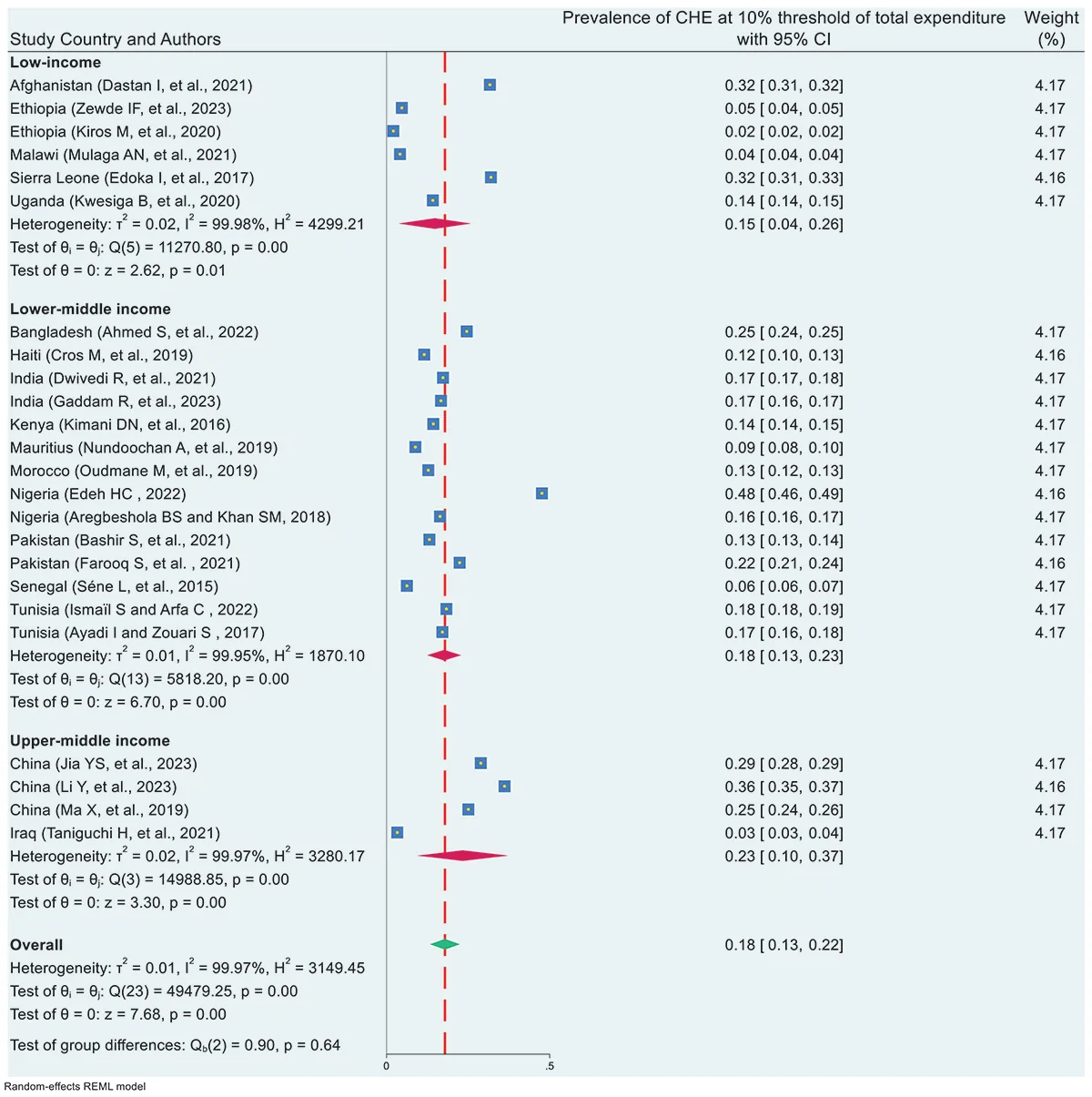
Results of subgroup analysis of CHE prevalence at the 10% of household’s total expenditure.

**Figure 4.**
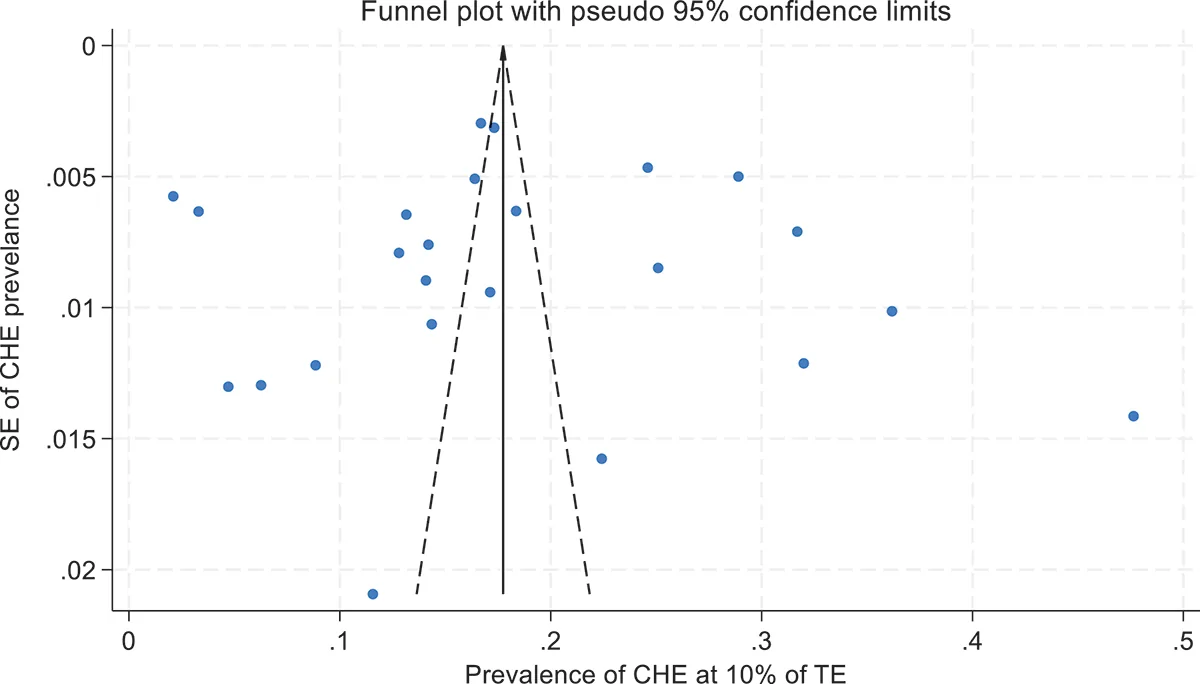
Funnel plot for assessing publication bias for the studies estimated CHE prevalence at the 10% of household’s total expenditure (TE).

### Prevalence of CHE at 40% threshold of the household’s non-food expenditure

Two studies by Mulaga et al. [39,40] from Malawi reported CHE prevalence, measured at a 40% threshold for non-food expenditure, using the same data set and yielding the same estimates. To avoid duplicates, we excluded CHE data from one study [40] and estimated the pooled CHE prevalence across studies of LICs, Lower-MICs, and UMICs at the 40% threshold using data from 32 studies. Again, the pooled prevalence estimate of 7% (95% CI: 5.0–10.0) (Figure 5) indicated a significant financial burden, with a lower estimate and narrower confidence interval than the estimate at 10% of the household’s total expenditure. Results also revealed considerable heterogeneity among studies $\left(I^{2}>99\%,\tau^{2}=0.01,p\;<\;0.001\right)$ and strong evidence of publication bias (bias coefficient = 48.41, *p* < 0.001), which is further supported by Funnel plot asymmetry (Figure 6). Again, the DOI plot with an LFK index of 4.24 exhibited major asymmetry (Figure S2 in the supplementary file), providing consistent evidence of publication bias. Sub-group analysis showed the pooled estimates in LICs (6 studies of 3 countries) [19,26,38–40,82] as 3.0% (95% CI: −1.0–7.0), in Lower-MICs (20 studies of 12 countries) [20,21,25,28,31–35,37,41–43,45,46,50,83–85] as 8.0% (95% CI: 4.0–12.0) and in UMICs (7 studies of 4 countries) [22–24,36,49,52,81] as 8.0% (95% CI: 3.0–12.0).

**Figure 5.**
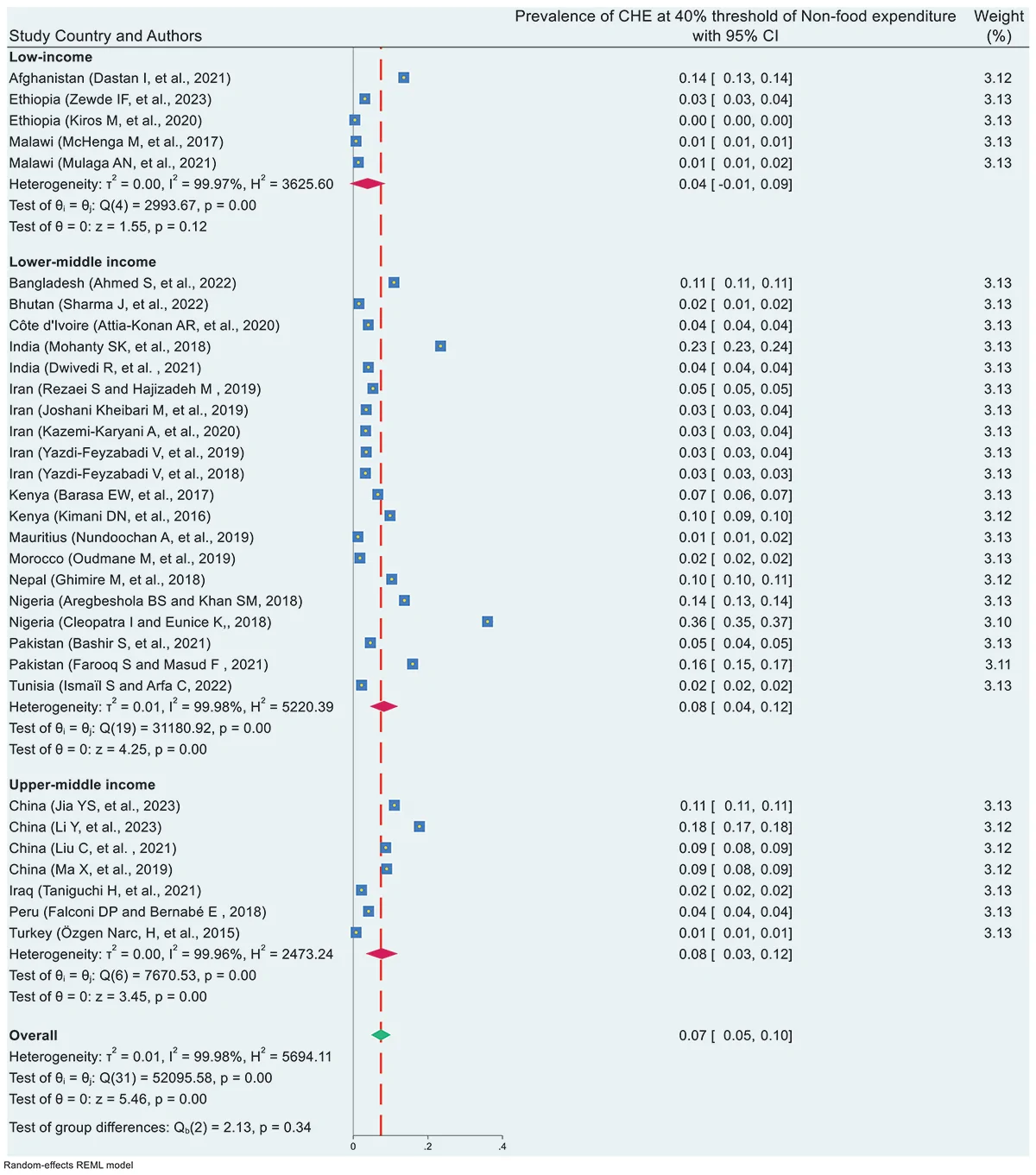
Results of subgroup analysis of CHE prevalence at the 40% of household’s non-food expenditure.

**Figure 6.**
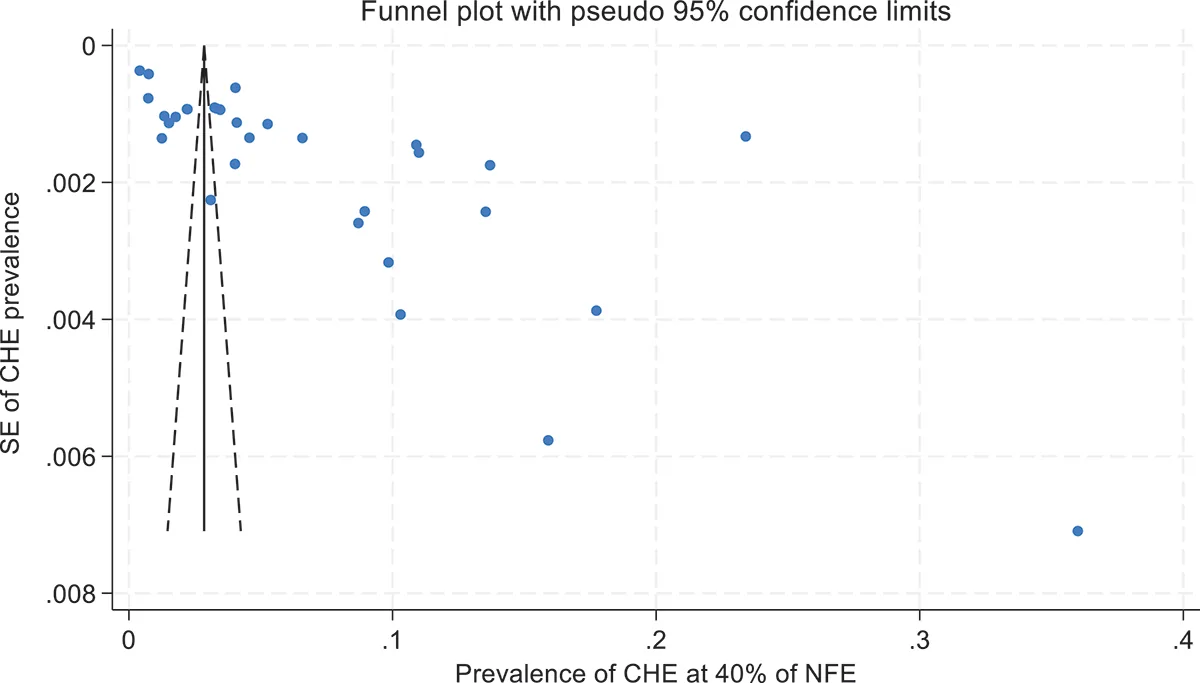
Funnel plot for assessing publication bias for the studies estimated CHE prevalence at the 40% of household’s non-food expenditure (NFE).

### Meta-regression results

To explore the source of heterogeneity, we further conducted multivariable random-effects meta-regression analyses for both the 10% and 40% thresholds using study-level characteristics, including publication year, income group, and study quality as covariates (Table 4). At both thresholds, none of the covariates were significantly associated with CHE prevalence (all *p* > 0.05), and the models explained none of the between-study heterogeneity (R^2^ = 0.0%). Results also exhibited that considerable residual heterogeneity remained (I^2^ > 99%) for both models.

**Table 4.** Multivariable random-effects meta-regression of CHE prevalence at the 10% threshold of total expenditure and the 40% threshold of non-food expenditure.

Covariate	CHE 10% Threshold		CHE 40% Threshold		
	β (95% CI)	p-value	β (95% CI)	p-value	
Publication year	0.011 (−0.012, 0.034)	0.362	0.001 (−0.012, 0.015)	0.853	
Lower-middle income vs Low-income	0.044 (−0.087, 0.176)	0.509	0.030 (−0.052, 0.111)	0.473	
Upper-middle income vs Low-income	0.071 (−0.082, 0.224)	0.365	0.041 (−0.049, 0.131)	0.372	
High quality vs Moderate quality	0.015(−0.110, 0.140)	0.814	−0.040 (−0.100, 0.021)	0.202	
**Model-level Statistics**					
**Model**	**Studies (n)**	**τ^2^**	**I^2^ (%)**	**R^2^ (%)**	**Wald χ^2^ (*p*-value)**
Model for CHE at 10% threshold	24	0.0141	99.95	0.00	2.31 (0.679)
Model for CHE at 40% threshold	32	0.0061	99.98	0.00	2.90 (0.574)

Low-income and moderate quality served as the reference categories; β, meta-regression coefficient; CI, confidence interval; τ^2^, between-study variance; I^2^, proportion of total variability attributable to heterogeneity; R^2^, proportion of between-study variance explained.

### Sensitivity analysis: latest study for each country

In our main meta-analysis (Figures 3 and 5), some countries contributed multiple studies. When countries with multiple relatively extreme-prevalence studies are included, they may disproportionately weight the pooled estimate, particularly in the presence of considerable heterogeneity (I^2^ > 99%). To estimate the robustness of our primary findings and investigate the potential influence of including multiple studies from a single country, we conducted a sensitivity analysis by using only the most recent study from each country. This approach minimizes within-country clustering and ensures uniform contribution from each country to the pooled effect estimate. Findings from the sensitivity analyses are displayed with forest plots (Figures 7 and 8).

**Figure 7.**
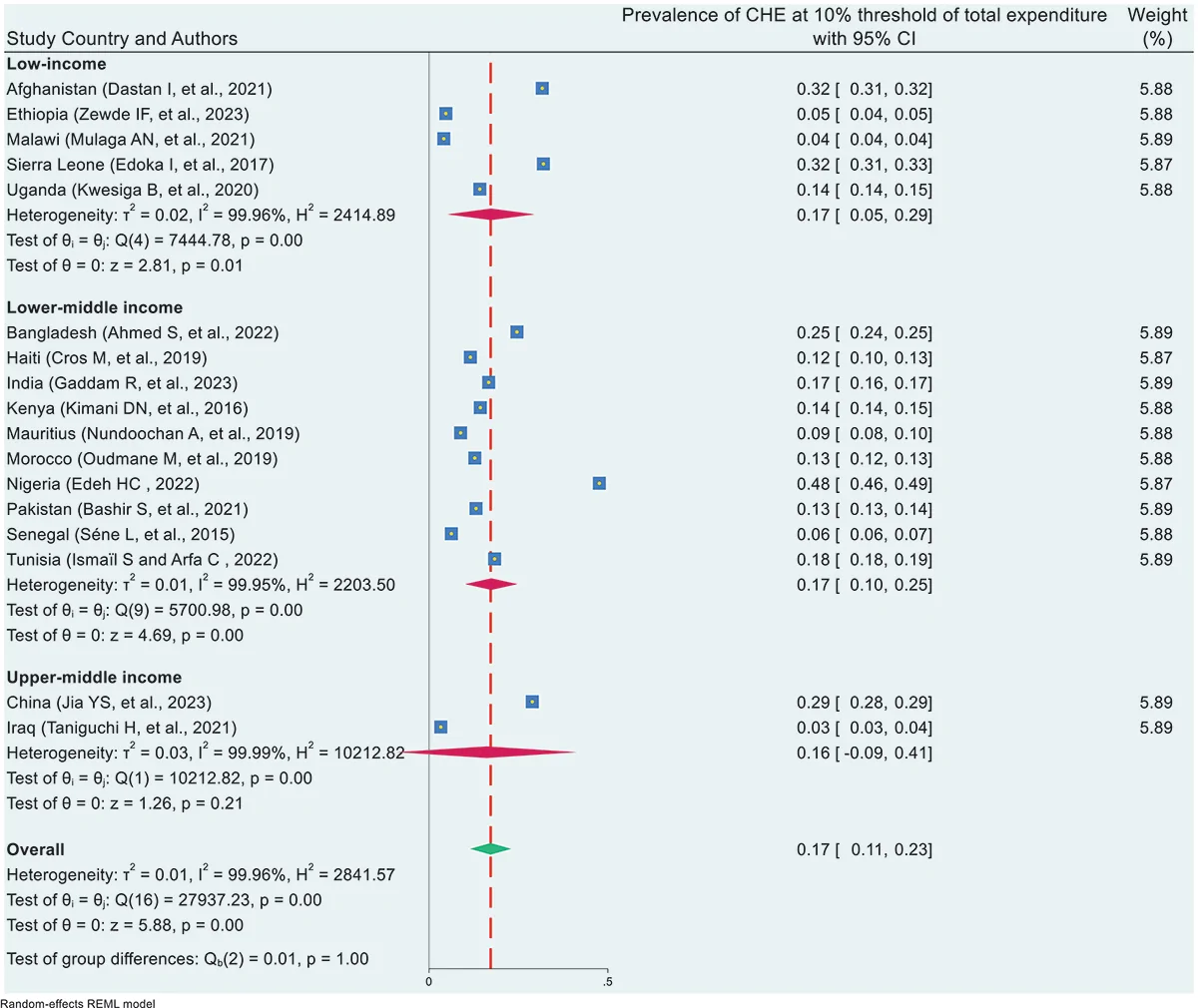
Results of sensitivity analysis of CHE prevalence at the 10% of household’s total expenditure (one study per country).

**Figure 8.**
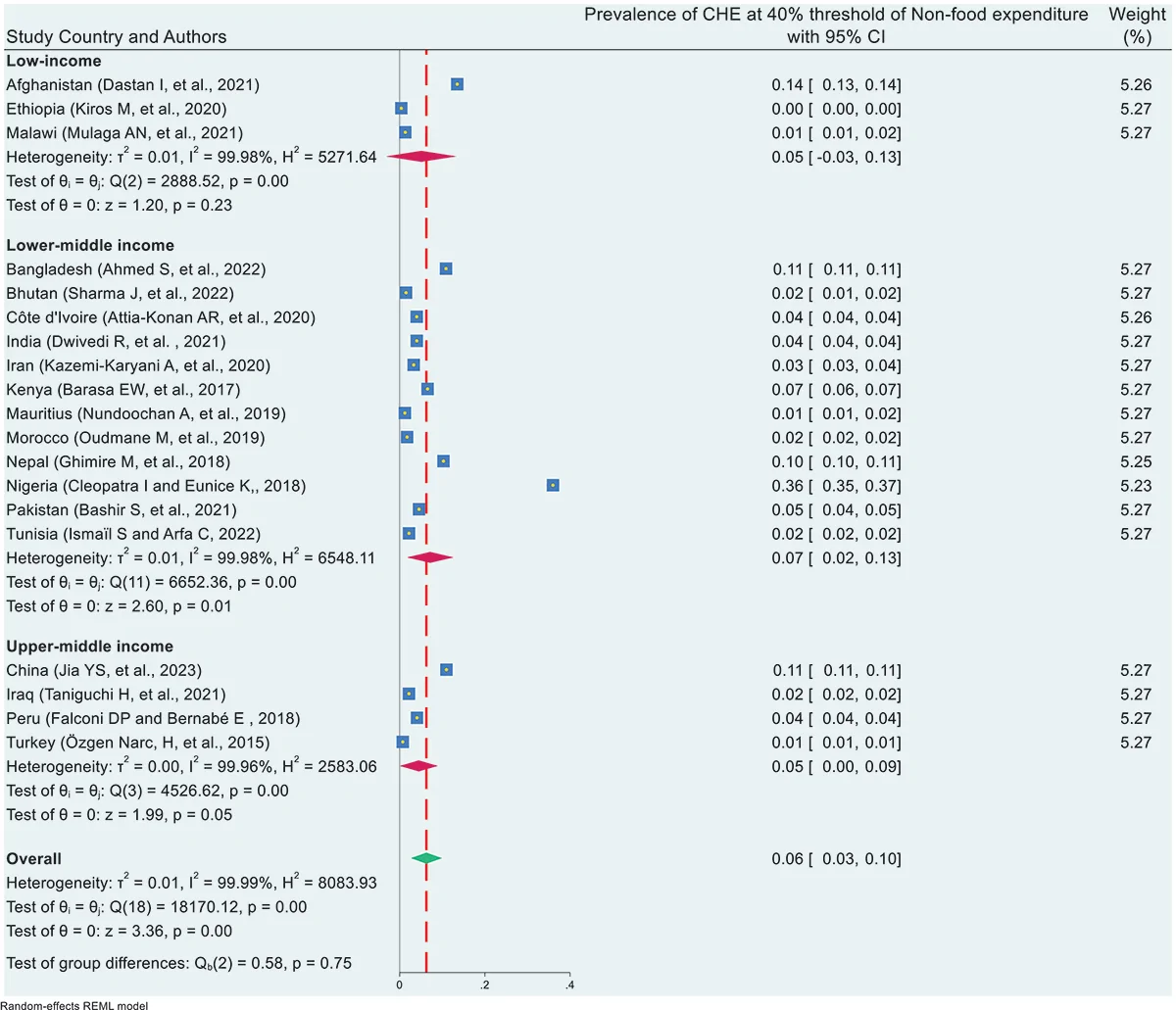
Results of sensitivity analysis of catastrophic health expenditure prevalence at the 40% of household’s non-food expenditure (one study per country).

At the 10% threshold, the pooled CHE prevalence estimate was 17% (95% CI: 11%–23%) (Figure 7), which is very close to the estimate from the full sample. Subgroup estimates were 17% (95% CI: 5%–29%) in LICs, 17% (95% CI: 10%–25%) in Lower-MICs, and 16% (95% CI: −9%–41%) in UMICs. In contrast to the primary analysis (LIC: 15%, Lower-MIC: 18%, UMIC: 23%), the subgroup estimates were slightly decreased, specifically for UMICs. However, the pooled effect size and direction remained consistent, suggesting that the pooled estimate was not substantially driven by the CHE measures for countries with multiple studies.

At the 40% threshold, the pooled estimate of CHE prevalence was 6% (95% CI: 3%–10%) (Figure 8), compared with 7% in the main analysis. Subgroup estimates were 5% (95% CI: −3%–13%) in LICs, 7% (95% CI: 2%–13%) in Lower-MICs, and 5% (95% CI: 0%–9%) in UMICs. These findings closely aligned with the main analysis (LIC: 3%, Lower-MIC: 8%, UMIC: 8%), suggesting that the influence of estimates from countries with multiple studies on pooled estimates was modest.

Overall, the sensitivity analyses confirm the stability of the main findings, which are not significantly changed by the inclusion of several studies from any single country.

### Certainty of evidence

The overall certainty of evidence for pooled estimates, assessed using the GRADE approach, was rated as low for both the 10% and 40% CHE thresholds (Table 5). Although the included studies were of moderate-to-high quality and directly addressed the review question, the evidence was downgraded due to between-studies heterogeneity (I^2^ > 99%) and evidence of publication bias.

**Table 5.** Grade assessment of certainty of evidence for pooled prevalence estimates of catastrophic health expenditure (CHE).

Outcome	No. of Studies	Risk of Bias	Inconsistency	Indirectness	Imprecision	Publication Bias	Overall Certainty (GRADE)	Rationale for Rating
CHE prevalence at 10% threshold of total household expenditure	24	Not serious	Very serious	Not serious	Not serious	Serious	⨁⨁◯◯ Low	Downgraded one level for considerable heterogeneity and one level for publication bias
CHE prevalence at 40% threshold of non-food expenditure	33	Not serious	Very serious	Not serious	Not serious	Serious	⨁⨁◯◯ Low	Downgraded one level for considerable heterogeneity and one level for publication bias

## Discussion

This systematic review and meta-analysis contributed important insights into both the determinants and CHE prevalence across LICs, Lower-MICs, and UMICs. This review included English-language studies that estimated CHE prevalence and associated factors using either of two common definitions: the 10% threshold of total household expenditure or the 40% threshold of no-food expenditure. Findings on the factors of CHE were synthesized narratively following a conceptual framework of six broad themes. Results of household head characteristics show that Female-headed households, especially in Lower-MICs, were more vulnerable to CHE. This is probably due to lower income and asset ownership, which support the evidence that gender differences in income and resource access worsen financial risk protection [99]. A few studies in this review found a lower risk of CHE among Female-headed households, again in Lower-MICs, which may be attributed to women’s tendency to avoid access and spend less on healthcare due to their generally weaker financial position compared to their male counterparts. Older household heads also faced a higher CHE risk, which may be due to their increased healthcare needs and limited earning capacity [60]. Conversely, higher educational attainment among household heads had a protective effect on CHE prevalence in LICs, Lower-MICs, and UMICs. Generally, people with higher education tend to be more aware of health and take more preventive measures. Moreover, they are likely to have a decent, more stable job and be able to cover their healthcare costs. This result is consistent with studies by Ashour and Adisa [100,101]. Like education, employment was also a protective factor, reducing CHE prevalence by improving financial capacity with permanent earnings and access to insurance [100–102].

Several household-level factors, including area of residence, income quintile, and household size, were associated with CHE risk but showed distinct patterns across income groups. The evidence on the association between residence and CHE prevalence was mixed, but rural disadvantage was more consistently associated with higher CHE risk, particularly in Lower-MICs and UMICs, while a few studies reported higher CHE prevalence in urban settings. These findings are in line with outcomes in countries across Southeast Asia, Sub-Saharan Africa, and South America [103–105]. Rural populations tend to have lower incomes, less education, and need to travel long distances because public healthcare services are more readily available in urban areas. These differences may affect the chance of facing CHE among rural households. Additionally, urban areas usually have more private healthcare facilities, where healthcare costs are generally higher than those in rural areas. Higher socioeconomic status and better living conditions were generally protective, particularly in Lower-MICs and UMICs, indicating greater financial resilience among wealthier households. The association between household size and CHE prevalence was also mixed, suggesting that CHE’s influence depends on household composition, dependency burden, and income-sharing arrangements. Household health expenditure, including inpatient and outpatient expenditures, has emerged as a prominent factor of CHE in Lower-MICs and UMICs, reflecting higher utilization of formal healthcare services and rising treatment costs as health systems expand.

Among household member characteristics, health-related needs emerged as the most consistent factor of CHE across income groups and thresholds. The presence of an elderly person and chronic illness are major CHE drivers across all income groups, particularly in Lower-MICs and UMICs. This is because older people use more healthcare services than younger people, and chronic illnesses require specialized care with long-term treatment. This result is consistent with studies of sub-Saharan Africa and other studies [103,104]. Consistent association of higher CHE with disabilities in Lower-MICs and UMICs, and hospitalization in LICs and Lower-MICs, reflecting greater healthcare needs and long-term treatment cost. The presence of children in the household showed mixed effects on CHE across LICs and Lower-MICs, suggesting that its effect may depend on household composition and local healthcare contexts.

Social protection factors, particularly health insurance coverage and public health expenditure, were generally associated with a lower risk of CHE, although their effects varied across income groups. The mixed effects of insurance status in Lower-MICs suggest that insurance coverage alone may be insufficient to protect households from CHE due to poor coverage or large copayments [106]. The consistently protective effects observed in UMICs indicate that higher levels of public health financing with no user fees can strengthen financial risk protection and shield households from CHE [41].

Health system factors also played an important role in protecting households from CHE. The use of private healthcare facilities is a main contributing factor to the increasing prevalence of CHE in Lower-MICs. Access to public healthcare services in LICs is very limited, and due to higher user fees, the cost of accessing private healthcare facilities is very high, given their socioeconomic conditions. Despite many improvements in healthcare facilities, people still face obstacles to accessing public healthcare and experience financial risk from CHE when accessing private healthcare facilities [107,108]. Additionally, other studies have found that using public health facilities is associated with a higher prevalence of CHE. This association may stem from the fact that specialized services in public hospitals are often costlier than in private facilities, increasing financial burden on households [85]. Longer distances to health facilities were also associated with higher CHE in Lower-MICs, reflecting persistent non-medical out-of-pocket costs and geographical barriers to access. Conversely, the present result showed that access to NGO-run or community-based health centers helped reduce CHE, particularly in Lower-MICs. The association between sociocultural factors and CHE was highly context-specific and was confined to Lower-MICs. The heterogeneous associations for region and social group affiliation suggest that their influence on CHE varies with social stratification, cultural practices, healthcare-seeking behavior, and economic opportunities.

This review also estimated the pooled prevalence of CHE in LMICs. Overall, 18% (95% CI: 14.0–23.0) and 7% (95% CI: 5.0–10.0) of households in LMICs faced CHE, estimated using thresholds of 10% and 40%, respectively. The finding is consistent with the study by Wagstaff et al. (2018), which reported a little lower CHE prevalence (11.7%) at the 10% threshold of total consumption, maybe due to differences in the number of included countries or time periods [4]. Findings are also aligned with the World Bank and WHO’s 2021 global monitoring reports [16], which emphasize that healthcare costs push millions of people into poverty every year.

The study observed considerable heterogeneity (I^2^ > 99%) in both pooled estimates, justifying the use of random-effects models in the meta-analysis. This high level of heterogeneity is common in global health financing studies and may be because of differences in survey design, recall periods, and health system contexts, as well as socio-cultural factors, including women’s access to paid labor, the availability of financial protection mechanisms, and the adequacy of retirement policy. Similarly, Eze et al. (2022) reported substantial variability in CHE estimations in sub-Saharan Africa due to differences in health service use, insurance coverage, and cost-sharing provision [17]. Thus, given the considerable heterogeneity (I^2^ > 99%), pooled prevalence estimates should be used with caution for policymaking in any single country; local estimates are essential.

Subgroup meta-analysis by income group revealed differences in CHE prevalence. At 10% threshold of household total expenditure, the pooled CHE prevalence was highest in UMICs (23%), followed by Lower-MICs (18%) and LICs (15%). At the 40% threshold of non-food expenditure, similarly, LICs reported the lowest CHE prevalence (3%), compared with 8% in both Lower-MICs and UMICs. The consistently lower CHE prevalence observed in LICs at both thresholds may reflect inequalities in healthcare access and unmet need, rather than improved financial risk protection. Households in LICs often limit their healthcare utilization due to economic and geographic barriers, thereby underestimating CHE incidence and hiding the true extent of financial risk, as previously stated by Kruk et al. (2018) [109]. In contrast, the higher CHE prevalence in UMICs suggests that economic growth may inconsistently increase CHE prevalence by expanding the scope for greater healthcare utilization, often through private or higher-cost providers, even in relatively better healthcare systems. This aligns with Xu et al. (2003), who highlighted that increasing national income does not essentially enhance FRP without targeted policy interventions [6]. This review found a very high prevalence of CHE (47.64%) in Nigeria among Lower-MICs, moderately high in China (36.18%) among UMICs, and in Sierra Leone (32%) among LICs, estimated using the 10% threshold.

Publication bias was confirmed jointly by Egger’s regression test, funnel plot asymmetry, and a DOI plot with the LFK index for both 10% and 40% thresholds, indicating that smaller studies were more likely to be published. This publication bias may affect the validity of our pooled estimates in two ways. First, it may lead to overestimation of the true CHE prevalence if studies with lower prevalence were unpublished or underreported. Second, publication bias may reduce the representativeness of the evidence base by underrepresenting studies reporting lower CHE prevalence, which could affect the accuracy of pooled estimates and limit their generalizability across the diverse contexts of LICs, Lower-MICs, and UMICs. Thus, this publication bias highlights the need for careful interpretation of pooled estimates and calls for a broad range of studies, particularly in LICs, where data may be lacking and could skew the estimates.

The meta-regression findings indicate that the considerable heterogeneity in CHE prevalence was not explained by publication year, income group, or study quality at either 10% or 40% thresholds. This suggests that other contextual and methodological factors, such as variations in survey design, recall periods, healthcare system characteristics, or socio-cultural contexts, may account for the high between-study heterogeneity.

A country-level sensitivity analysis with one study per country showed consistent pooled estimates at both thresholds. The overall prevalence at the 10% threshold stayed unaffected (18%), whereas at the 40% threshold, it shifted slightly from 7% to 6%. Although small shifts were observed in subgroup estimations, mainly in UMICs, from 18% to 16% and 8% to 5%, at the 10% threshold and 40% threshold, respectively, the overall conclusions remained unchanged (18%) at 10% threshold, but a small shift from 6% to 5% at 40% threshold. This indicates that including multiple studies from countries did not significantly bias the pooled estimates. This method in systematic reviews recommends testing the dependence of results on individual studies [110], thereby increasing the trustworthiness of our findings. It indicates that, even with multiple studies from the same country and considerable heterogeneity, the overall magnitude and direction of CHE prevalence remain stable in LICs, Lower-MICs, and UMICs. These findings are consistent with global monitoring reports [16,111], which underscore that gaps in FRP persist and comprehensive policy programs are still needed.

The GRADE assessment indicated low-certainty evidence for the pooled prevalence estimates, primarily due to considerable heterogeneity and publication bias. Therefore, the pooled estimates should be interpreted as summary measures of CHE prevalence across diverse settings rather than as precise estimates for individual countries. However, the consistency of findings across subgroup analyses, meta-regression, and sensitivity analyses supports the robustness of study findings.

This systematic review has several key strengths. It follows a comprehensive, rigorous methodology that adheres to PRISMA guidelines and a registered protocol (CRD42024521553), ensuring transparency. The study uses a comprehensive search strategy across numerous databases and manual searches, covering literature from 30 September 2015 to 30 November 2023, ensuring the time span and the evidence’s relevance to the SDGs. This review makes a novel contribution to the existing literature on CHE prevalence by synthesizing fragmented evidence from 40 studies across LICs, Lower-MICs, and UMICs, thereby providing a robust, comprehensive perspective on CHE prevalence and its associated factors, which were not captured in previous studies. By assessing factors across income classes, it not only highlights consistent factors associated with CHE prevalence but also reveals context-specific differences in CHE drivers that reflect differences in socioeconomic and healthcare system structures. These findings advance prior research by underlining how individual and household socioeconomic factors intersect with healthcare inequities. From a policy perspective, the review provides valuable insights for designing more equitable health financing strategies, including targeted subsidies, expanded insurance coverage, and regulated private-sector pricing, to strengthen financial risk protection and advance progress toward UHC.

Despite this review’s strengths, certain limitations should be considered in interpreting its findings. Firstly, restricting inclusion to English-language studies, considering the 2015–2023 period, and relying on household survey data may introduce selection bias. Studies published in non-English languages and those published after November 2023 could add to and provide up-to-date evidence on CHE prevalence. Secondly, this review captured the prevalence and determinants of financial risk from CHE using only the thresholds of 10% of a household’s total expenditure and 40% non-food expenditure. Synthesis of studies that reported results using other thresholds could be considered, as we may have missed some insights in this review. Thirdly, unmet healthcare needs are more severe in LICs, Lower-MICs, and UMICs [112], yet the associated costs were not captured in this review. Incorporating CHE estimates that considered unmet healthcare needs could have provided a more comprehensive and accurate assessment of CHE prevalence. Fourthly, considerable heterogeneity among studies, due to significant variations in CHE prevalence and CHE threshold definitions, may limit the comparability of studies and the generalizability of the results. Consequently, the pooled estimates should be interpreted as a central tendency across highly diverse settings, not as a precise prediction for any specific country. Finally, this study considered only quantitative but excluded qualitative studies. If qualitative studies could be included, the acceptability and generalizability of this review would have increased.

## Conclusion

This review suggests that area of residence, socioeconomic position, the presence of an aged household member, household members with chronic illness, and the use of private healthcare facilities are the factors most consistently associated with CHE prevalence in LICs, Lower-MICs, and UMICs. However, these factors are deeply rooted in wider social, political, and economic contexts, underscoring the need for deeper inquiry into how individual-level risk factors interact with structural conditions such as social policies, governance, and power relations beyond the clearer influences like healthcare system design. To achieve financial risk protection under UHC for the SDGs, policymakers should consider those factors to reduce health inequality while designing healthcare financing mechanisms. The findings underscore the need for comprehensive reforms of the health financing system that address both demand-side barriers (through expanded insurance coverage, targeted subsidies for the elderly, members with chronic illnesses, female-headed and rural households, and regulated private sector pricing to prevent exploitative healthcare) and supply-side constraints (through improved healthcare access and quality). By adopting evidence-based policies tailored to local contexts, governments of LICs, Lower-MICs, and UMICs can make meaningful progress toward UHC and financial protection for all citizens.

## Supplementary Material

Supplementary file_R1.docx

PRISMA_2020_checklist_R1.docx

## Data Availability

This study uses data from the published articles listed in this review. All data are available within this review and its supplementary file. Anyone can access this data for free.
