# Supplementary material for: Catastrophic health expenditure and associated factors in low-, lower-middle and upper-middle-income countries: systematic review and meta-analysis
Source: Glob Health Action. 2026 Sep 25;19(1):2734678. doi: 10.1080/16549716.2026.2734678 (PMC13618086; doi:10.1080/16549716.2026.2734678)
Supplement: Supplementary file 2 [file ZGHA_A_2734678_SM2846.docx]

Table S1. Search string

|  | **String** | **CINAHL** | **PubMed** | **Scopus** | **Web of Science** |
| --- | --- | --- | --- | --- | --- |
| 1 | (“influencing factors” OR “factors influencing” OR “determinants” OR “determining factors” OR “influential factors” OR “contributing factors” OR “factors contributing” OR “factors associated” OR “associated factors”) | All text | All text | Title, Abstract, Keyword | Title, Abstract, Keyword (called "Topic") |
| 2 | ("financial protection" OR "financial risk" OR “financial crisis” OR "financial distress" OR "Catastrophic" OR "Catastrophe" OR "Catastrophes" OR "Catastrophically" | All text | All text | Title, Abstract, Keyword | Title, Abstract, Keyword, (called "Topic") |
| 3 | ("health" OR "healthcare" OR "health care" OR "health uptake" OR "health wellbeing" OR "healthcare utilization" OR "healthcare utilisation" OR "healthcare access") | All text | All text | Title, Abstract, Keyword | Title, Abstract, Keyword, (called "Topic") |
| 4 | ("expenditure" OR "cost*" OR "payment*") | All text | All text | Title, Abstract, Keyword | Title, Abstract, Keyword, (called "Topic") |
| 5 | (Afghanistan OR Albania OR Algeria OR Angola OR Argentina OR Armenia OR Azerbaijan OR Bangladesh OR Belarus OR Belize OR Benin OR Bhutan OR Bolivia OR "Bosnia And Herzegovina" OR Botswana OR Brazil OR Bulgaria OR "Burkina Faso" OR Burundi OR "Cabo Verde" OR Cambodia OR Cameroon OR "Central African Republic" OR Chad OR China OR Colombia OR Comoros OR "Democratic Republic Of Congo" OR Congo OR "Costa Rica" OR "Côte d'Ivoire" OR Cuba OR Djibouti OR Dominica OR "Dominican Republic" OR Ecuador OR Egypt OR "El Salvador" OR "Equatorial Guinea" OR Eritrea OR Eswatini OR Ethiopia OR Fiji OR Gabon OR Gambia OR Georgia OR Ghana OR Grenada OR Guatemala OR Guinea OR "Guinea-Bissau" OR Haiti OR Honduras OR India OR Indonesia OR Iran OR Iraq OR “Ivory Coast” OR Jamaica OR Jordan OR Kazakhstan OR Kenya OR Kiribati OR "Republic Of Korea" OR Kosovo OR Kyrgyz* OR "Lao" OR Lebanon OR Lesotho OR Liberia OR Libya OR Macedonia OR Madagascar OR Malawi OR Malaysia OR Maldives OR Mali OR Mauritania OR Mauritius OR Mexico OR Micronesia OR Moldova OR Mongolia OR Montenegro OR Morocco OR Mozambique OR Myanmar OR Namibia OR Nepal OR Nicaragua OR Niger OR Nigeria OR Pakistan OR Palau OR "Papua New Guinea" OR Paraguay OR Peru OR Philippines OR Russia* OR Rwanda OR Samoa OR “São Tomé And Príncipe” OR Senegal OR Serbia OR "Sierra Leone" OR "Solomon Islands" OR Somalia OR "South Africa" OR "Sri Lanka" OR "Saint Lucia" OR "Saint Vincent And The Grenadines" OR Sudan OR Suriname OR "Syria*" OR Tajikistan OR Tanzania OR Thailand OR Timor-Leste OR Togo OR Tonga OR Tunisia OR Turkey OR Türkiye OR Turkmenistan OR Tuvalu OR Uganda OR Ukraine OR Uzbekistan OR Vanuatu OR Venezuela OR Vietnam OR "West Bank And Gaza Strip" OR Yemen OR Zambia OR Zimbabwe | Title / Abstract | Title / Abstract | Title / Abstract | Title / Abstract |
| 6 | 1 AND 2 AND 3 AND 4 AND 5 |  |  |  |  |

Table S2: Eligibility criteria and grouping of the studies included in the review

| **Study Inclusion Criteria** | | |
| --- | --- | --- |
| **Study Characteristics** | Perspective | Studies that explicitly reported the prevalence and/or determinants of catastrophic health expenditure (CHE), either as a primary outcome or alongside related measures such as out-of-pocket expenditure or impoverishment |
|  | Setting | Studies conducted for or focused on low-, lower-middle-, and upper-middle-income countries. It will adopt the current World Bank income classification for countries (per capita Gross National Income (GNI) for low-income countries is US$ 1,085 or less; for lower-middle-income countries is between US$ 1,086 and US$ 4,255; for upper-middle-income countries is between US$ 4,256 and US$ 13,205). |
|  | Time/Timing | Since the Sustainable Development Goals (SDGs) with the target 3.8 to achieve UHC, including coverage of healthcare services (SDG 3.8.1) and financial risk protection (SDG 3.8.2) by the United Nations in 2015, the time frame for the literature search is 2015 to 2023. To reflect the increased global attention to financial risk protection following the WHO World Health Report 2010, studies were eligible if the underlying household survey data were collected in 2010 or later. |
| **Report Characteristics** | Research Design | Studies with a quantitative design. |
|  | Outcome | Studies explicitly quantified the prevalence of CHE either at 10% threshold of household’s total expenditure or 40% of capacity to pay (commonly operationalized as non-food expenditure) from cross-sectional population level surveys for general healthcare and identified associated factors |
|  | Language | Due to time limitations, only studies published in English were considered. |
|  | Publication status | Reports published in peer-reviewed journals indexed in online databases. The review will also consider reports documented in the reference list of identified studies from databases as grey literature. |
| **Grouping characteristics of the included studies for synthesis** | | The studies will be grouped for synthesis based on the following–  Income class –low, lower-middle, and upper-middle-income countries |
| **Exclusion Criteria** | | |
| **Language and Geographic location** | | Non-English studies will be excluded |
| **Setting** | | Studies from the High-Income Countries will not be considered |
| Research Design | | Studies with a qualitative design. |
| **outcome** | | Prevalence of CHE measured using a threshold other than 10% threshold of the household’s total expenditure or 40% threshold of non-food expenditure. Studies that measured CHE prevalence based on household survey data collected before 2010 were excluded. |

Table S3. AXIS tool for Risk of Bias Assessment

| Item | | Yes / No / Don’t Know/ Comment |
| --- | --- | --- |
| Introduction | |  |
| 1 | Were the aims/objectives of the study clear? |  |
| Methods |  |  |
| 2 | Was the study design appropriate for the stated aim(s)? |  |
| 3 | Was the sample size justified? |  |
| 4 | Was the target/reference population clearly defined? (Is it clear who the research was about?) |  |
| 5 | Was the sample frame taken from an appropriate population base so that it closely represented the target/reference population under investigation? |  |
| 6 | Was the selection process likely to select subjects/participants that were representative of the target/reference population under investigation? |  |
| 7 | Were measures undertaken to address and categorise non-responders? |  |
| 8 | Were the risk factor and outcome variables measured appropriate to the aims of the study? |  |
| 9 | Were the risk factor and outcome variables measured correctly using instruments/measurements that had been trialled, piloted or published previously? |  |
| 10 | Is it clear what was used to determined statistical significance and/or precision estimates? (eg, p values, CIs) |  |
| 11 | Were the methods (including statistical methods) sufficiently described to enable them to be repeated? |  |
| Results |  |  |
| 12 | Were the basic data adequately described? |  |
| 13 | Does the response rate raise concerns about non-response bias? |  |
| 14 | If appropriate, was information about non-responders described? |  |
| 15 | Were the results internally consistent? |  |
| 16 | Were the results for the analyses described in the methods, presented? |  |
| Discussion | |  |
| 17 | Were the authors’ discussions and conclusions justified by the results? |  |
| 18 | Were the limitations of the study discussed? |  |
| Other |  |  |
| 19 | Were there any funding sources or conflicts of interest that may affect the authors’ interpretation of the results? |  |
| 20 | Was ethical approval or consent of participants attained? |  |

Table S4. Determinants of the prevalence of financial risk from CHE at 10% threshold of Household’s total expenditure

| **Determinants of financial risk from CHE at 10% of the household’s total expenditure** | **Increase Prevalence of CHE** | | | | **Decrease Prevalence of CHE** | | | |
| --- | --- | --- | --- | --- | --- | --- | --- | --- |
|  | **LICs** | **Lower-MICs** | **UMICs** | **Total** | **LICs** | **Lower-MICs** | **UMICs** | **Total** |
| **Household Head characteristics** |  |  |  |  |  |  |  |  |
| Female (reference=male) | 1 |  | 2 | 3 |  | 3 |  | 3 |
| Age (Continuous) | 2 | 1 |  | 3 |  | 1 |  | 1 |
| Literacy(reference=Illiterate) |  |  |  |  |  | 3 |  | 3 |
| Education (ref.=No formal education) |  |  |  |  |  | 1 |  | 1 |
| Primary |  |  |  |  | 1 | 1 | 1 | 3 |
| Middle |  |  |  |  |  |  |  |  |
| Secondary |  | 1 |  | 1 | 1 | 1 | 1 | 3 |
| University |  |  |  |  |  | 1 | 1 | 2 |
| Marital Status (reference= never married) | 1 |  |  | 1 |  | 1 |  | 1 |
| Employment (ref.=unemployed) |  |  |  |  | 1 | 4 |  | 5 |
| Head of the household is retired |  | 1 |  | 1 |  |  |  |  |
| **Household characteristics** |  |  |  |  |  |  |  |  |
| Area of residence (reference=Urban) | 2 | 5 | 3 | 10 |  | 2 |  | 2 |
| Socioeconomic status (reference = Poorest) |  |  |  |  |  | 1 |  | 1 |
| 2nd | 1 | 1 | 1 | 3 |  | 4 | 2 | 6 |
| 3rd | 1 | 1 | 1 | 3 |  | 4 | 2 | 6 |
| 4th | 1 | 1 | 1 | 3 |  | 4 | 2 | 6 |
| Richest | 2 | 1 | 1 | 4 |  | 5 | 1 | 6 |
| Poverty (reference= non-poor) |  |  |  |  | 1 |  |  | 1 |
| Household size | 3 | 2 |  | 5 |  | 4 | 1 | 5 |
| Insurance status (reference =Yes) |  | 1 |  | 1 |  | 2 |  | 2 |
| Number of persons per room |  | 1 |  | 1 |  |  |  |  |
| Having a toilet facility (reference = no) |  |  |  |  |  | 1 |  | 1 |
| Electricity for lighting (Reference= Kerosene/oil/candle) |  |  |  |  |  | 1 |  | 1 |
| Cooking sources (ref. coal/fire/dung/charcoal) |  |  |  |  |  |  |  |  |
| Gas |  |  |  |  |  | 1 |  | 1 |
| No arrangement |  |  |  |  |  | 1 |  | 1 |
| Household food expenditure per capita |  |  |  |  |  |  | 1 | 1 |
| Household total expenditure |  | 1 |  | 1 |  |  |  |  |
| Inpatient service expenses |  | 1 | 1 | 2 |  |  |  |  |
| Outpatient expenses |  | 1 |  | 1 |  |  |  |  |
| Households consume alcohol and tobacco |  | 1 |  | 1 |  |  |  |  |
| Economic Shock in the household | 1 |  |  | 1 |  |  |  |  |
| **Household members Characteristics** |  |  |  |  |  |  |  |  |
| Presence of children | 1 | 3 |  | 4 |  | 1 |  | 1 |
| Presence of an elderly person | 1 | 9 | 2 | 12 | 1 |  |  | 1 |
| Presence of disable member |  | 1 |  | 1 | 1 |  |  | 1 |
| Presence of a hospitalized member | 1 | 1 |  | 2 |  |  |  |  |
| Chronic illness in a household member | 2 | 3 |  | 5 |  |  |  |  |
| Infectious and parasitic diseases | 1 |  |  | 1 |  |  |  |  |
| Circulatory system and endochronic diseases | 1 |  |  | 1 |  |  |  |  |
| Experience with severe illness/injury |  |  |  |  |  |  |  |  |
| Accident in household member |  | 1 |  | 1 |  |  |  |  |
| Respiratory disease | 1 |  |  | 1 |  |  |  |  |
| Number of sick days |  |  |  |  |  |  |  |  |
| **Social Protection Characteristics** |  |  |  |  |  |  |  |  |
| Insurance status (reference =Yes) |  | 1 |  | 1 |  | 2 |  | 2 |
| Public-funded health expenditure (% of GDP) |  |  |  |  |  |  | 1 | 1 |
| **Health System Characteristics** |  |  |  |  |  |  |  |  |
| Utilization of public facility | 1 | 2 |  | 3 |  |  |  |  |
| Utilization of private facility |  | 2 |  | 2 |  |  |  |  |
| Utilization of NGO facility |  |  |  |  |  | 1 |  | 1 |
| Utilization of other facility (reference=No) |  | 1 |  | 1 |  |  |  |  |
| Utilized private facility (reference=Public) | 1 |  |  | 1 |  |  |  |  |
| Use of religious health facility (Ref.=public) |  | 1 |  | 1 |  |  |  |  |
| Distance to the health facility |  | 1 |  | 1 |  |  |  |  |
| Utilization of Health Center |  |  |  |  |  | 1 |  | 1 |
| Utilization of Health Post |  |  |  |  |  | 1 |  | 1 |
| **Sociocultural Factors** |  |  |  |  |  |  |  |  |
| Religion (reference = Hindu) |  |  |  |  |  |  |  |  |
| Muslim |  | 1 |  | 1 |  |  |  |  |
| Christian |  | 1 |  | 1 |  |  |  |  |
| Others (Sikhism, Jainism etc.) |  | 1 |  | 1 |  | 1 |  | 1 |
| Social group (reference = General) |  |  |  |  |  |  |  |  |
| Tribe |  |  |  |  |  | 1 |  | 1 |
| Cast |  |  |  |  |  | 1 |  | 1 |
| Others |  |  |  |  |  | 1 |  | 1 |

*LICs: Lower-income countries; Lower-MICs: Lower-middle-income countries; UMICs: Higher-income countries*

Table S5. Determinants of the prevalence of financial risk from CHE at 40% threshold of non-food expenditure

| **Determinants of financial risk from CHE at 40% of Non-food expenditure** | **Increase Prevalence of CHE 40%** | | | | **Decrease Prevalence of CHE 40%** | | | |
| --- | --- | --- | --- | --- | --- | --- | --- | --- |
|  | **LICs** | **Lower-MICs** | **UMICs** | **Total** | **LICs** | **Lower-MICs** | **UMICs** | **Total** |
| **Household Head characteristics** |  |  |  |  |  |  |  |  |
| Gender (reference =male) | 1 | 5 | 1 | 7 |  | 2 |  | 2 |
| Age |  | 5 | 1 | 6 | 1 |  |  | 1 |
| Literate (reference =Illiterate) |  |  |  |  |  | 5 | 1 | 6 |
| Education (reference =No formal education) |  |  |  |  |  | 2 |  | 2 |
| Primary |  |  |  |  | 1 | 1 |  | 2 |
| middle school |  |  |  |  |  |  | 1 | 1 |
| Secondary |  |  |  |  | 1 | 2 |  | 3 |
| University |  |  |  |  | 1 | 3 |  | 4 |
| Marital Status |  |  |  |  |  |  |  |  |
| Married |  | 2 | 1 | 3 |  |  |  |  |
| Employment (reference=unemployed) |  |  |  |  | 1 | 5 | 1 | 7 |
| Head of the household is retired |  | 1 |  | 1 |  |  |  |  |
| Head of household being homemakers |  | 1 |  | 1 |  |  |  |  |
| **Household characteristics** |  |  |  |  |  |  |  |  |
| Area of residence Rural (reference =Urban) | 2 | 9 | 3 | 14 |  | 1 |  | 1 |
| Socioeconomic status (reference =Poorest) |  |  |  |  |  | 1 | 1 | 2 |
| 2nd | 1 | 1 |  | 2 | 1 | 6 | 2 | 9 |
| 3rd | 1 | 1 |  | 2 | 1 | 7 | 4 | 12 |
| 4th | 1 | 1 |  | 2 | 1 | 7 | 3 | 11 |
| Richest | 1 | 1 |  | 2 | 1 | 8 | 3 | 12 |
| Household size | 1 | 2 |  | 3 | 1 | 6 | 4 | 11 |
| Number of persons per room |  | 1 |  | 1 |  |  |  |  |
| Having a toilet facility |  |  |  |  |  | 1 |  | 1 |
| Electricity for lighting (Ref.= Kerosene/oil/candle) |  |  |  |  |  | 1 |  | 1 |
| Cooking sources(reference=Coal/fire/dung/charcoal) |  |  |  |  |  |  |  |  |
| Gas |  |  |  |  |  | 1 |  | 1 |
| Others |  |  |  |  |  |  |  |  |
| No arrangement |  |  |  |  |  | 1 |  | 1 |
| Household food expenditure per capita |  |  |  |  |  |  | 1 | 1 |
| Household with more commodities |  |  |  |  |  | 1 |  | 1 |
| Inpatient service expenses |  | 2 | 2 | 4 |  |  |  |  |
| Outpatient expenses |  | 2 |  | 2 |  |  |  |  |
| Economic Shock in the household | 1 |  |  | 1 |  |  |  |  |
| **Household members Characteristics** |  |  |  |  |  |  |  |  |
| Presence of children |  | 2 |  | 2 |  | 3 |  | 3 |
| Presence of elderly person |  | 13 | 3 | 16 |  | 1 |  | 1 |
| Presence of disabled member | 1 | 2 | 1 | 4 |  |  |  |  |
| Presence Hospitalized member | 1 | 3 |  | 4 |  |  |  |  |
| Chronic illness in a household member |  | 6 | 1 | 7 |  |  |  |  |
| Infectious and parasitic diseases | 1 |  |  | 1 |  |  |  |  |
| Circulatory system and endochronic diseases | 1 |  |  | 1 |  |  |  |  |
| Experience with severe illness/injury |  | 1 |  | 1 |  |  |  |  |
| Respiratory disease | 1 |  |  | 1 |  |  |  |  |
| Dental care used |  | 1 |  | 1 |  |  |  |  |
| **Social Protection Characteristics** |  |  |  |  |  |  |  |  |
| Insurance status (reference=Yes) |  | 2 | 2 | 4 |  | 1 |  | 1 |
| Publicly funded healthcare expenditure (% of Total Health expenditure) |  |  |  |  |  |  | 1 | 1 |
| **Health system factors** |  |  |  |  |  |  |  |  |
| Utilization public facility |  | 1 |  | 1 |  |  |  |  |
| Utilization of private facility |  | 1 |  | 1 |  |  |  |  |
| Utilization of the NGO facility |  |  |  |  |  | 1 |  | 1 |
| Utilization of other facility (reference = no) |  | 1 |  | 1 |  |  |  |  |
| Utilized private facility (reference=Public) |  | 1 |  | 1 |  |  |  |  |
| Use of religious health facility (reference=public) | 1 |  |  | 1 |  |  |  |  |
| Distance to the healthcare facility |  | 1 |  | 1 |  |  |  |  |
| **Sociocultural Factors** |  |  |  |  |  |  |  |  |
| Religion (reference = Hindu) |  |  |  |  |  |  |  |  |
| Muslim |  |  |  |  |  |  |  |  |
| Christian |  |  |  |  |  |  |  |  |
| Others |  |  |  |  |  | 1 |  | 1 |

*LICs: Lower-income countries; Lower-MICs: Lower-middle-income countries; UMICs: Higher-income countries*

Figure S1. DOI Plot for CHE Prevalence at 10% Threshold

Figure S2. DOI Plot for CHE Prevalence at 40% Threshold
